# Are there enough reasons to justify an early surgical approach in left-sided valve infective endocarditis? A retrospective analysis

**DOI:** 10.1186/1471-2334-13-S1-P91

**Published:** 2013-12-16

**Authors:** Valeriu Gheorghiță, Aida Răşcanu, Florin Alexandru Căruntu, Loredana Benea, Bogdana Manu, Olga Dorobăț, Doina Iovănescu, Adrian Streinu-Cercel

**Affiliations:** 1 National Institute for Infectious Diseases “Prof. Dr. Matei Balş”, Bucharest, Romania; 2 ”Dr. Carol Davila” Central Military Emergency University Hospital, Bucharest, Romania; 3 Carol Davila University of Medicine and Pharmacy, Bucharest, Romania

## Background

International guidelines provide strong recommendations about the indications of surgery for infective endocarditis. However, in clinical practice, there are a lot of parameters that need to be taken in consideration, such as: duration of prior antibiotic therapy, patient age, the presence of extracardiac complications or preexistent comorbidities.

## Methods

We performed a retrospective study over a period of four years (2007-2010) which included all patients diagnosed and treated for left-sided valve infective endocarditis (IE) in the National Institute for Infectious Diseases “Prof. Dr. Matei Balş”, Bucharest. The main objective of our study was assessing the pathological features and early outcomes of our patients managed conservatively in order to determine the best therapeutic approach.

## Results

We included 381 patients diagnosed with left-sided native and prosthetic valve infective endocarditis. The median age was 59 years and patients with native valve IE (75.8%) predominated. The interested valves (mitral and aortic) were similarly affected in patients with native and prosthetic valve IE (median value 39% [IQR, 38-40] vs. 36% [IQR, 32-40]).The percentage of blood culture negative IE was 56.1%. The indication for early surgery according to the international guidelines was strong in 32.2% of patients due to severe valvular dysfunction with severe heart failure (47.9%) and systemic embolism (30.8%). The in-hospital mortality rate was 7.6% (n=29) but we could not assess the early mortality rate after patient discharge.

## Conclusion

Our data demonstrated that there are enough reasons for early surgical approach of patients with IE, considering the poor outcomes in the absence of surgery.

